# Dexmedetomidine Sedation in Dogs: Impact on Electroencephalography, Behavior, Analgesia, and Antagonism with Atipamezole

**DOI:** 10.3390/vetsci11020074

**Published:** 2024-02-06

**Authors:** Jeff C. Ko, Carla Murillo, Ann B. Weil, Matthia Kreuzer, George E. Moore

**Affiliations:** 1 College of Veterinary Medicine, Purdue University, West Lafayette, IN 47907, USA; murilloc@purdue.edu (C.M.); aweil@purdue.edu (A.B.W.); gemoore@purdue.edu (G.E.M.); 2 School of Medicine, Technical University of Munich, 80333 Munich, Germany; m.kreuzer@tum.de

**Keywords:** dexmedetomidine, spindle waves, constant rate of infusion, dogs, EEG, cardiorespiratory, analgesia, bradycardia, atipamezole, antagonism

## Abstract

**Simple Summary:**

This study investigated the application of electroencephalography (EEG) to monitor dexmedetomidine-induced sedation and its subsequent reversal with atipamezole in dogs. EEG effectively traced alterations in brain activity, indicating reduced responsiveness during sedation and a return to normal patterns upon reversal. Dexmedetomidine CRI induced deep sedation, immobilizing the dog with noticeable bradycardia and hypertension during the CRI phases. Atipamezole successfully reversed the sedative effects, aligning both EEG and cardiovascular parameters with baseline values. These findings suggest the potential clinical utility of EEG-guided sedation and atipamezole-antagonized dexmedetomidine, warranting further research for method refinement and optimal application across diverse clinical scenarios.

**Abstract:**

This study aimed to assess the impact of dexmedetomidine constant rate infusion (CRI) on key parameters in dogs. Six dogs received a 60 µg/kg/h dexmedetomidine infusion over 10 min, followed by three 15 min decremental CRIs (3, 2, and 1 µg/kg/h). A subsequent reversal phase employed 600 µg/kg/h atipamezole over 5 min. Continuous electroencephalogram (EEG) assessment, and cardiorespiratory and analgesia monitoring (every 3 min) were conducted, including analgesia evaluation through responses to electric stimulation. Dexmedetomidine induced profound sedation, evidenced by lateral recumbency and immobility. Patient State Index (PSI) decreased from awake (90.4 ± 4.3) to Phase 1 (50.9 ± 30.7), maintaining sedation (29.0 ± 18.1 to 33.1 ± 19.1 in Phases 2–4). Bradycardia (37.8 ± 3.5 bpm, lowest at Phase 3) and hypertension (133.7 ± 17.0 mmHg, highest at Phase 1) were observed, with minimal analgesia. Atipamezole promptly reversed sedation, restoring cognitive function (tail wagging behavior), and normalizing cardiovascular parameters. During atipamezole CRI, the EEG exhibited a transition from delta waves to alpha and low beta waves. This transition was observed alongside gradual increases in PSI and electromyographic activities. Additionally, spindle activities disappeared during this process. This study’s results suggest potential clinical utility for EEG-guided dexmedetomidine sedation with reversal using atipamezole, warranting further investigation.

## 1. Introduction

Dexmedetomidine, a highly selective α2-adrenoreceptor agonist, functions as a sedative and analgesic for animal and human anesthesia, given either as a single dose or via continuous infusion [1,2,3,4,5,6,7,8,9], with typical doses ranging from 0.5 to 3 mcg/kg/h [1,2,3,4,5,6,7,8,9].

Dexmedetomidine’s sedative effects stem from its targeted binding to α2-adrenergic receptors in the locus coeruleus, a brainstem region governing wakefulness. This binding inhibits norepinephrine release, a crucial neurotransmitter for alertness, effectively dampening neuronal activity in brain areas associated with vigilance [1,8,9,10,11]. This disrupted thalamocortical connectivity underpins dexmedetomidine’s sedative properties [8,9,10,11]. Beyond sedation, dexmedetomidine also modulates cardiovascular, respiratory, and pain pathways through interaction with α2-adrenoreceptors in various brainstem nuclei [1,7,8,9,10,11]. In dogs, it initially triggers peripheral vasoconstriction, causing a transient rise in blood pressure, followed by a baroreflex-mediated decrease in heart rate and cardiac output [1,2,3,5,6,7]. Additionally, dexmedetomidine’s analgesic properties arise from activating α2-adrenoreceptors in specific brainstem regions, alleviating pain perception [8,11].

Atipamezole is an α2-adrenergic receptor antagonist. It competitively inhibits α2-adrenergic receptors and rapidly antagonizes the sedative and analgesic effects induced by dexmedetomidine and medetomidine in dogs [1,3,12,13,14]. Intramuscular administration of atipamezole is frequently used, and the reversal effect is usually rapid. The median arousal time after atipamezole to antagonize medetomidine (40 mcg/kg, IM) was 3–5 min, and walking time was 6–10 min compared to >30 min for both effects after placebo [14]. The literature review reveals limited information on the intravenous use of atipamezole in dogs. In a previous study, atipamezole was administered at 200 mcg/kg intravenously 30 min after 40 mcg/kg medetomidine intravenously in dogs [15]. The result concluded that atipamezole reversed medetomidine-induced vasoconstriction with a significant decrease in diastolic blood pressure and increased oxygen supply to peripheral tissues as indicated by a lower tissue oxygen-extraction ratio. Very few studies have been conducted using atipamezole as a continuous rate infusion (CRI) to reverse dexmedetomidine-induced sedation in dogs.

Electroencephalography (EEG) is a non-invasive method that has been used to assess the depth of sedation and general anesthesia in human patients [9,10,11,16]. EEG measures the electrical activity of the brain and can provide information about the level of consciousness, including patients under dexmedetomidine sedation. This information can be used to guide the administration of sedatives and ensure the appropriate depth of sedation is achieved [9,10,11,16,17,18].

Recent advancements in EEG monitoring technology have sparked interest in using EEG to monitor the depth of sedation and general anesthesia along with various other clinical applications in animals, including dogs [19,20,21,22,23,24,25,26]. It seemed logical to evaluate the sedation induced by dexmedetomidine with a constant infusion to see if the modern EEG monitor can be used to characterize the EEG pattern changes of the dog under different levels of sedation. Furthermore, the changes in the various dexmedetomidine dosages associated with the EEG characteristics, cardiorespiratory values, and its antagonism with atipamezole in dogs could also be explored.

The objectives of this study aimed to investigate the effects of dexmedetomidine CRI on sedation, EEG patterns, cardiorespiratory function, pain perception, and behavior changes in dogs. Additionally, we explored the impact of using atipamezole with CRI to counteract dexmedetomidine CRI-induced sedation, focusing on EEG patterns, cardiovascular changes, and behavioral alterations induced by dexmedetomidine CRI. Our hypothesis was that EEG can effectively track the depth changes in dexmedetomidine CRI-induced sedation, alongside cardiovascular changes. Furthermore, we anticipated that EEG could detect atipamezole’s antagonistic effects on dexmedetomidine-induced sedation and brain state changes.

## 2. Materials and Methods

### 2.1. Animals

Six healthy male research beagles were used in this descriptive experimental study. They were all 16 months old and weighed between 10 and 13 kg. Prior to the study, the beagles underwent health assessment, including a physical examination, complete blood count, serum chemistry analysis, and urinalysis.

On the day of anesthesia, the beagles were fasted for 8 h but had free access to water. The study protocol and the use of research animals were approved by the Purdue University Animal Care and Use Committee. The research animals were closely monitored in terms of their use and care, along with facilities and experimental designs. This monitoring ensures compliance with federal regulations and alignment with the three Rs endorsed by the Institutional Animal Care and Use Committee, ultimately confirming the ethical nature of the research.

### 2.2. Experimental Design and Treatment Timeline (Table 1)

On the day of the study, a cephalic venous catheter was placed in each dog. The CRI dosages in this study were derived from our pilot study results. The dexmedetomidine CRI solution was made by mixing 0.2 mL of dexmedetomidine (Dexdomitor^®^, Zoetis Inc., Kalamazoo, MI, USA) with 9.8 mL of physiological saline, resulting in a total volume of 10 mL at a concentration of 10 μg/mL. It was administered using a Medfusion^®^ syringe pump (Model 3500 V6, Smiths Medical, St. Paul, MN, USA).

For the atipamezole CRI solution, the total amount needed was calculated at 50 µg/kg. The calculated volume was withdrawn from the original drug solution (Antisedan^®^, Zoetis Inc., Kalamazoo, MI, USA) and then diluted with sterile sodium chloride 0.9% to achieve a total volume of 5ml. This volume was delivered using the Medfusion^®^ syringe pump (Model 3500 V6, Smiths Medical, St. Paul, MN, USA) at the rate specified in the study. Both infusions were administered through an IV extension set directly attached to the preplaced IV catheter. 

The awake baseline of the vital signs (see below) and EEG were recorded before the sedation started. Thereafter, the sedation procedure commenced with an initial intravenous dose of 10 μg/kg (or 60 μg/kg/h) dexmedetomidine administered over 10 min. This sedation was maintained through three decremental CRI doses, each lasting 15 min (Table 1). All infusions were controlled using a syringe pump, with dosages sequentially decreasing as follows: 3 μg/kg/h, 2 μg/kg/h, and finally, 1 μg/kg/h. The complete duration of the study when dexmedetomidine was administered was 55 min. Following the last dexmedetomidine CRI administration, the dogs promptly received atipamezole treatment through an intravenous dose of 50 μg/kg administered over 5 min (or 600 μg/kg/h).

**Table 1:** Treatment protocol and timeline.

	Drug Treatment and Dosage	Duration of CRI	Assessments *
0	None (Awake measurements)	N/A	EEG, ECG, CR
1	Dexmedetomidine (60 μg/kg/h)	10 min	EEG, ECG CR every 3 min
2	Dexmedetomidine CRI (3 μg/kg/h)	15 min	EEG, ECG, CR and Nox every 3 min
3	Dexmedetomidine CRI (2 μg/kg/h)	15 min	EEG, ECG, CR and Nox every 3 min
4	Dexmedetomidine CRI (1 μg/kg/h)	15 min	EEG, ECG, CR and Nox every 3 min
5	Atipamezole (600 μg/kg/h)	5 min	EEG and ECG CR every 3 min
6	None (Recovery period)	Until sternal recumbency achieved	EEG, ECG, CR every 3 min Awake state and recovery assessment

* Continuous monitoring involved EEG (electroencephalogram) and ECG (electrocardiogram). CR represents cardiorespiratory function, and Nox signifies the evaluation of the response to noxious stimuli.

The treatment protocol and timeline details are provided in Table 1, in addition to the continuous collection of EEG data every 2 s by the EEG monitor throughout the entire experiment. Analgesic properties were assessed (see below) by application of noxious stimuli (NOX) as indicated in Table 1. Furthermore, cardiorespiratory assessments were conducted at intervals of every 3 min within each phase of the experiment.

### 2.3. Monitoring the EEG, Cardiorespiratory, Analgesic, and Behavioral Parameters

The dogs’ brain state changes during the study were continuously monitored using frontal electrodes and the Sedline^®^ EEG monitor (Masimo Corporation, Irvine, CA 92618, USA) [26]. To adapt the Sedline^®^ adult adhesive electrodes for use on dogs with varying skull sizes, these electrodes were affixed to six standard EEG needle electrodes using alligator clips. This modification ensured consistent placement [26]. Prior to each study, the electrode system underwent testing for proper impedance, and all six electrodes had to pass the Sedline monitor’s impedance test to ensure accurate EEG information.

Baseline vital signs (blood pressure, heart rate, and electrocardiogram) were measured with a multi-parameter monitor. Cardiovascular assessment included continuous Lead II ECG monitoring for cardiac arrhythmias and oscillometric blood pressure measurements every 3 min using a blood pressure cuff (at a size of 40% of the limb circumference) on one hindlimb. SpO_2_ was measured continuously using a lingual probe on the dog’s tongue. The pulse rate (heart rate) was determined by simultaneously palpating the femoral pulse and auscultating the heart. The respiratory rate of the dog was evaluated through visual observation of its thoracic excursion during the study. Throughout the study, the dog was positioned on either side in lateral recumbency and remained in that position until the dog recovered and started moving. The rectal temperature was monitored every 6 min, and the body temperature was maintained using towels covering the body. If necessary, a forced hot air blanket was employed to keep the body temperature within the range of 38.0 °C to 39.0 °C. During the study, balanced electrolyte was administered at a rate of 10 mL/kg/hour through an infusion pump, using the same IV catheter to ensure both the dog’s hydration and the patency of the catheter for CRI drug administration.

Analgesia was assessed during sedation maintenance using electrical stimulation via two 22-gauge needles inserted 5 cm apart into the lateral tibia [26]. The nerve stimulator was programmed in tetanus mode to deliver a 0.22 ms square wave pulse stimulus at 400 V, with intensity settings ranging from 0 to 100 Hz [26]. Stimulation was administered every 3 min after physiological data collection, starting at the lowest setting and lasting 2 s. Analgesic assessment was excluded during Phases 1, 5, and 6 (Table 1) due to the light plane of sedation in the treated dogs.

Purposeful movement (limb withdrawal, head or neck motion, or tail or nose twitching) was defined as a positive behavioral response to electrical stimulation [26]. Stimulation intensity was increased upon lacking a positive response or until the maximum level was reached. 

During the CRI phases, behavioral signs linked with dexmedetomidine, such as immobilization, head and tongue drooping, and non-response to vocal stimulation, were observed and documented for each dog. In the atipamezole reversal and recovery phases, any awakening behaviors or signs of excitation, such as vocalization, excessive muscle activity, tail wagging, or paddling were carefully noted and recorded. Although the sedative behavior was observed, it is important to note that no specific subjective sedation score was assigned due to the non-blinded study design.

### 2.4. EEG and Cardiorespiratory Data Analysis

The processed EEG data were continuously monitored and automatically saved as CSV files by the Sedline monitor, which included Patient State Index (PSI), Burst Suppression Ratio (SR), electromyography (EMG) activity, 95% Spectral Edge Frequency (SEF95) for both hemispheres of the brain, and artifact (ART) activity. The raw EEG data recordings in edf format were downloaded for visual inspection [26]. 

Statistical analysis was carried out on the CSV files that contained 2 s processed EEG data for each dog for the entire experiment, resulting in over 20,000 data points for specific variables. SEF95 data were computed separately for the left and right hemispheres, and hemispheric coherence was assessed using paired *t*-tests.

For the EMG activity, the Sedline monitor uses electrodes on the forehead to measure muscle activity in the face and forehead. This activity includes facial movements like grimacing, jaw clenching, and eye blinking. The Sedline monitor then uses this information to calculate a PSI value, which scores how sedated and relaxed the dog is [26].

For the density spectral array (DSA), raw EEG data from the Sedline monitor was resampled to 89 Hz to address a known technical issue [26]. DSA creation was executed using MATLAB’s pwelch function with a 0.35 Hz frequency resolution, based on 5-s EEG segments with a 1 s shift.

The analysis involved the examination of the entire experimental EEG (every two seconds dataset) and cardiorespiratory data (every three minutes) within each experimental phase. For EEG parameters (PSI, SR, SEF95, EMG, and ART) and cardiorespiratory parameters (pulse/heart rate, respiratory rate, hemoglobin oxygen saturation, systolic arterial blood pressure, diastolic arterial blood pressure, and mean arterial blood pressure) for each dog, data grouping by experimental phase was performed. Mean values were compared using linear mixed model analyses with repeated measurements, and statistical significance was determined at *p* < 0.05. In cases where specific variables exhibited overall significant differences among phases, pairwise comparisons were made with Bonferroni correction for multiple comparisons. Data presentation included mean ± SD for both hemodynamic and EEG indices. 

## 3. Results

### 3.1. Dexmedetomidine Effects during the Initial Dose Phase of CRI (Phase 0–1)

**Figure 1:**
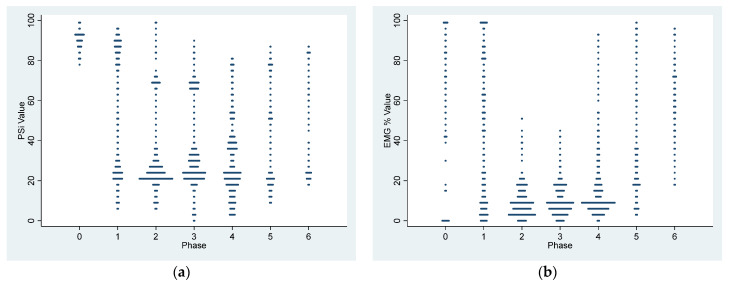
Dot plot presentation of the mean Patient State Index (**a**) and electromyography (**b**) of the six study dogs in response to dexmedetomidine and atipamezole infusion. Both PSI and EMG were initially high during the awake state and the initial dose of dexmedetomidine CRI (Phase 0–1). During the dexmedetomidine CRI sedation maintenance stage (Phase 2–4), both PSI and EMG decreased until atipamezole CRI and recovery (Phase 5–6), as shown in the dot plots.

**Figure 2:**
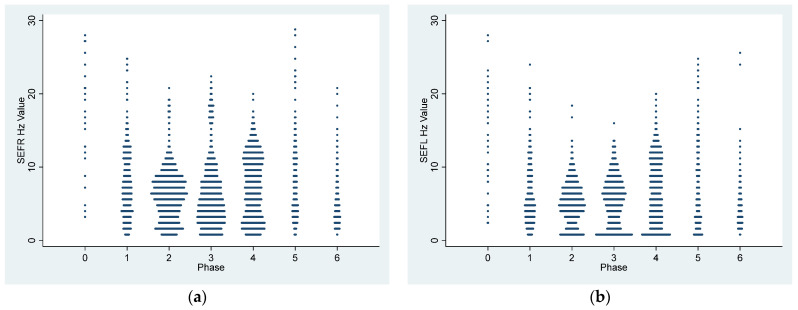
Dot plot distribution of mean spectral edge frequency 95 of the right (**a**) and left (**b**) hemispheres of the brain of the six study dogs. SEF95 is a marker of brain activity and was measured from the awake state (Phase 0) to dexmedetomidine CRI (Phase 1–4), atipamezole CRI (Phase 5), and recovery (Phase 6), showing a clear transition of brain state from awake to dexmedetomidine-induced sedation, to atipamezole antagonism, and to complete recovery in both the right and left hemispheres.

**Figure 3:**
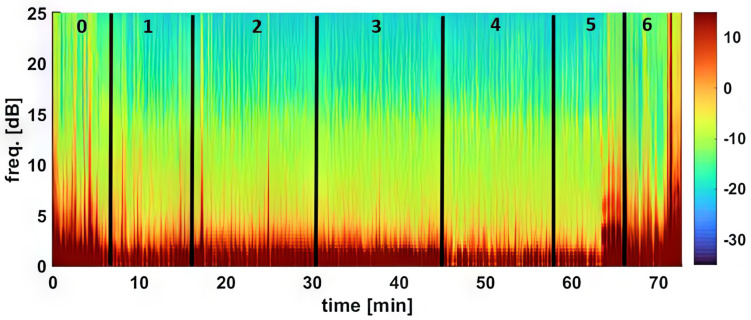
The density spectral array of a study dog subjected to dexmedetomidine CRI during the experimental phase. The median frequency of the EEG power is depicted on the Y-axis. The X-axis is the treatment time in minutes. The number in each treatment phase is listed on top of the figure, with 0 representing the awake phase, 1 representing the initial dose of dexmedetomidine, 2–4 representing the three decremental doses of dexmedetomidine constant rate of infusion maintenance, 5 representing atipamezole CRI, and 6 representing the recovery phase. The figure shows an increase in slow wave (0.1–4 Hz) power (density in the dark red color) from Phase 1 to peak at Phase 3, followed by a decrease in Phase 4. EEG power clearly shifted from Phase 4 to Phase 5 and 6 during the recovery.

**Figure 4:**
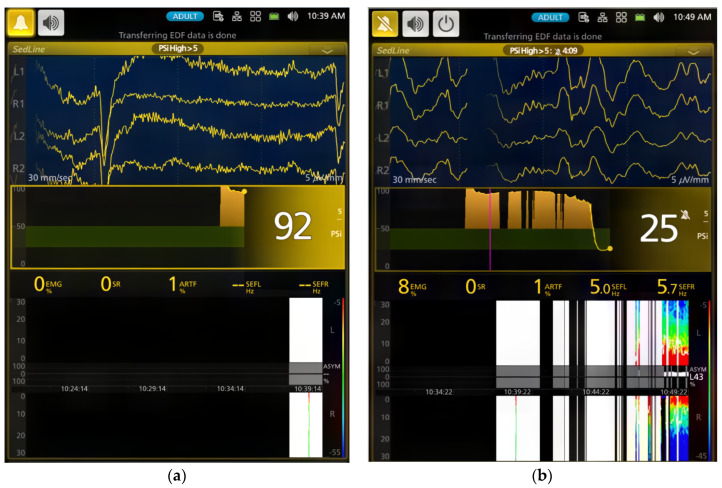
The figure shows a study dog’s awake EEG pattern (**a**—Phase 0), dominated by gamma waves (low amplitude, high frequency) with a PSI of 92. After dexmedetomidine CRI (**b**—Phase 1), the pattern changed to delta and theta waves with spindle waves, PSI decreased to 25, and SEF95 was 5.0–5.7 Hz.

**Figure 5:**
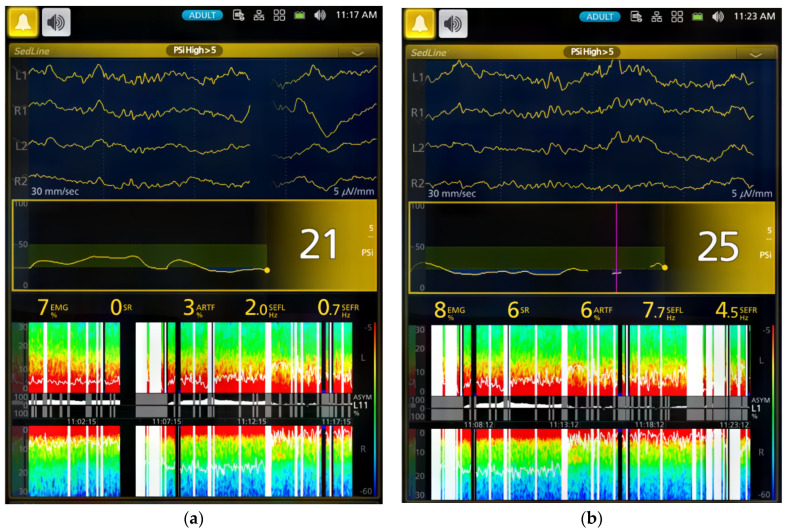
During the dexmedetomidine CRI maintenance phases Phase 2 (**a**) to Phase 4 (**b**), the EEG patterns were both slow waves (<1 Hz) and delta waves (1–4 Hz) with dominant spindle waves. PSI was stable between 21 and 25, EMG activity was low between 7 and 8%, and SEF95 was between 0.7 and 7.7 Hz. Spindle wave activity was well reflected in the DSA at a power of 8–13 Hz.

**Figure 6:**
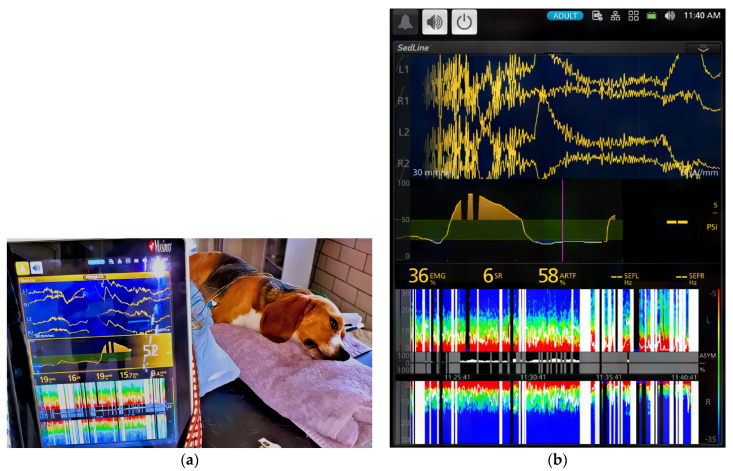
(**a**) During atipamezole CRI, the dog regained cognitive function with eyes open and tail wagging behavior. PSI increased to 52, SEF95 increased to alpha (9.4 Hz) and low beta (15.7 Hz) range and spindle waves were absent. EMG activity increased to 19%. (**b**) As the dog recovered further, EEG returned to the awake pattern, and both EMG (19% to 36%) and ART (19% to 58%) increased.

Table 2 and Table 3 and Figure 1 and Figure 2 summarize the EEG, cardiorespiratory, and analgesic data of six dogs during dexmedetomidine CRI in seven experimental phases. Figure 3, Figure 4, Figure 5 and Figure 6 depict a study dog, highlighting raw EEG, DSA patterns, and trends, along with processed EEG indices throughout each experimental phase.

At baseline, the dogs were awake and alert, with high-frequency EEG activity and strong muscle activity (Figure 1, Figure 3 and Figure 4). Dexmedetomidine-induced sedation was marked by head drooping, recumbency, and acceptance of a face mask for oxygen supplementation. This was coupled with lower-frequency EEG activity and reduced muscle activity. Additionally, there were observed changes in heart rate (decreased) and blood pressure (increased) associated with dexmedetomidine, as detailed in Table 3. Minimal changes in the respiratory rate were observed throughout the study. The dogs exhibited minimal analgesia, as they could be aroused by the increasing intensity of the noxious stimuli despite being under sedation.

### 3.2. Dexmedetomidine Effects during the Sedative Maintenance Phases (Phase 2–4)

During phases 2–4 of the sedation process, dogs displayed low PSI, signifying deep sedation. The highest SR was observed in Phase 4, while EMG activities remained consistently low. Additionally, there was a consistent presence of slow delta wave EEG activity (Table 1, Figure 1, Figure 2, Figure 3, Figure 4 and Figure 5). These processed EEG indices align with behavioral signs characterized by lateral recumbency and complete immobilization.

Figure 3 illustrates the DSA of EEG changes in a study dog during dexmedetomidine CRI. Spectrogram power notably increased in the slow delta wave (0.1–4 Hz) frequency band during CRI maintenance phases 2–4, signifying deep sedation. The power shift observed between Phase 3 and Phase 4, evident in both intensity color and pattern, became more pronounced in the latter part of Phase 5 and Phase 6, indicating a progressively lighter sedative state towards recovery. Additionally, as the dexmedetomidine dose was reduced in Phase 4, there was an increase in theta and alpha waves (Figure 2 and Figure 3).

Analgesic activity reached its peak in Phase 2 and declined in Phase 4, as indicated in Table 2. However, there was no significant difference observed in the analgesic activities elicited by the electrical stimulation. 

Spindles were frequently seen during the CRI, especially during Phase 4 maintenance (Figure 4 and Figure 5). Despite the EEG changes, heart rate, blood pressure, and respiratory rate remained stable, and the dogs displayed characteristic chemical immobilization behavior. The ECG assessment revealed sinus bradycardia without any observed heart blocks or other types of cardiac arrhythmias.

### 3.3. Effects of Atipamezole CRI on the Dexmedetomidine-Induced Effects during Recovery (Phase 5–6)

During atipamezole CRI, the dogs gradually resumed normal behavior, including tongue retraction and nociceptive response. Some dogs initially exhibited slow brain waves. Although the heart rate significantly increased compared to previous phases (Phase 1–4), it remained significantly lower than the baseline. Systolic blood pressure significantly decreased from Phase 1, and both mean and diastolic blood pressures significantly decreased from both Phase 1 and Phase 2. However, these blood pressures did not significantly differ from the baseline values. Signs of arousal with a return of cognition, such as tail wagging, head-lifting, and response to human voices, coincided with EEG pattern changes. All dogs smoothly recovered within five minutes following the conclusion of atipamezole CRI.

## 4. Discussion

In this study, we employed both processed EEG indices and raw EEG to evaluate changes in brain state associated with dexmedetomidine-induced sedation in healthy young adult dogs. Processed EEG parameters, like PSI, are used in both human patients and research dogs to monitor anesthetic-induced sedation depth [25,26,27,28]. PSI values range from 0 to 100, where a PSI of 0 indicates the deepest sedation, while 100 denotes an awake state. The recommended range for dexmedetomidine-sedated human patients typically falls between 25 and 50 [27]. Our findings indicate a substantial decline in PSI values over time during sedation. PSI significantly (*p* < 0.0001) decreased from 90.4 ± 4.3 in the awake state to 50.9 ± 30.7 during Phase 1 of dexmedetomidine CRI. Thereafter, the PSI substantially decreased to a range between 29.0 ± 18.1 and 33.1 ± 19.1 during sedative maintenance in Phases 2–4 (Table 2). Although the optimal sedative range of PSI for dogs is yet to be established, our study suggests that sedation was most profound during CRI maintenance phases (Phase 2–4), aligning with human sedative PSI values. 

Moreover, the changes in PSI recorded in this study effectively tracked the sedative and cognitive status changes in dogs, transitioning from the awake phase to sedative maintenance and returning to cognition following the administration of atipamezole during the recovery phase. This aligns with reports in dexmedetomidine-sedated humans, where BIS and EEG-based spectral entropy were utilized [27,28], underscoring the capability of these EEG-based indices to discern the depth of dexmedetomidine-induced sedation.

Different classes of anesthetic drugs induce distinct EEG patterns through neuro-oscillations [11,16]. These patterns persist as long as the drug remains in the central nervous system. By modulating these oscillations, the drugs impede effective communication between different parts of the brain, leading to the induction of sedation and anesthesia. The drug-specific nature of these neuro-oscillations serves as a unique identifier, essentially functioning as a fingerprint [11,16]. In humans, propofol exhibits different EEG oscillation patterns compared with dexmedetomidine [11,16]. The EEG patterns observed in dexmedetomidine-sedated dogs in the current study are characterized by slow-wave oscillations (5.1 ± 2.7 and 7.2 ± 4.2 Hz) along with spindle activities (8–13 Hz). These findings closely parallel those in humans, where dexmedetomidine induces EEG patterns similar to deep sleep, with slow-delta oscillations and spindle waves, resembling non-rapid eye movement sleep (NREM) stages II and III [8,10,11,16]. In essence, the EEG patterns in the dogs in this study closely resemble those seen in humans during dexmedetomidine sedation. Similar low-frequency and high-amplitude EEG wave bands were reported in dogs sedated with alpha-2 sedatives, including xylazine [21], medetomidine [22], and dexmedetomidine [22,23].

In dogs, dexmedetomidine is known to induce both hypertension and reflex bradycardia that is secondary to the hypertension [1,14]. Parallel to the dexmedetomidine-induced EEG changes, cardiovascular alterations are evident during the same time frame. Notably, blood pressure increases from the baseline, and reflex bradycardia due to such hypertension emerges following the initiation of dexmedetomidine CRI. These synchronous changes suggest that dexmedetomidine acts on target receptors in the brainstem, exerting effects on the cardiovascular center and inducing these impacts [1,14]. While dexmedetomidine elicits hemodynamic changes akin to those seen with a single dose [1,14], our current study reveals that a continuous infusion of dexmedetomidine leads to a gradual decrease in heart rate, intricately linked with a rise in blood pressure over time, as illustrated in Table 3.

It has been shown that dexmedetomidine administered through CRI at doses of 0.5, 1, and 3 µg/kg/hour has antinociceptive effects when used concurrently with isoflurane and other general-anesthetic drugs [29,30]. A recent comprehensive review [31], encompassing both human and animal (rat) studies, highlighted the challenges associated with assessing changes in the requirements of coadministered inhaled anesthetics in the presence of alpha-2 adrenergic agonists such as clonidine and dexmedetomidine. These challenges arise from the intricate interplay between their hypnotic and antinociceptive activities [31]. The complexity is further compounded when these alpha 2 adrenergic agents are administered alone, making it challenging to achieve their target analgesic effects [31], as observed in the current study. 

In the present study, the analgesic effect of dexmedetomidine CRI was not markedly pronounced, which allowed dogs to be aroused from their sedative state. This outcome underscores the premise that dexmedetomidine CRI alone, at the given dosage, produces a minimal analgesic effect. Particularly noteworthy is the understanding that true analgesia involves antinociceptive/analgesic activity which significantly contributes to the state of anesthesia/sedation by preventing nociceptive-induced arousal [31]. This emphasizes the sedative nature of dexmedetomidine and prompts reflection on its role as an anesthetic adjunct. 

The dogs in this study exhibited cognitive impairment during sedation, demonstrated by their acceptance of the face mask with a pulse oximeter sensor placed on their tongue, immobilization, and the inability to wag their tails. These behaviors were most pronounced during the dexmedetomidine maintenance phases (Phases 2–4). As tail wagging behavior in dogs involves complex emotional and cognitive brain functions [32], the regaining of tail wagging ability during atipamezole CRI, despite still being in lateral recumbency, suggests a shift in brain state, enabling the processing of information necessary for executing tail movements.

This study also explored the use of continuous atipamezole infusion to counteract CRI dexmedetomidine-induced sedation in dogs. The results demonstrated that atipamezole had a clinically relevant impact on both brain state and cardiovascular activity. As dogs gradually emerged from sedation, improvements were observed in alertness, normal tongue-withdrawal movement, and responsiveness to human presence with tail wagging activities.

However, initial variations in EEG patterns were noted, with two dogs exhibiting slower brain waves and others showing increased alpha and low beta activity. It is also interesting to note that the distinctive spindle wave activities characteristic of dexmedetomidine sedation disappeared in all dogs during the atipamezole CRI phase.

Atipamezole, typically approved for IM use to avoid potential cardiovascular side effects such as hypotension, has been reported in cats [33]. In a previous study [15] conducted on young healthy dogs, atipamezole was administered at 200 µg/kg IV to antagonize sedation induced by medetomidine at 40 µg/kg IV. Atipamezole reversed medetomidine-induced vasoconstriction and significantly reduced diastolic blood pressure (from 130 mmHg to 85 mmHg, *p* < 0.05) with an increase in heart rate, but minimally changed the systolic blood pressure in these treated dogs [15]. Both blood pressures were well within normal ranges in these atipamezole-treated dogs, with a reduction in tissue oxygen-extraction ratio, which signifies an increased oxygen supply to the peripheral tissue [15]. In the current study, atipamezole was administered as a CRI, instead of a bolus injection, so that a gradual change in the EEG pattern in these dexmedetomidine-sedated dogs could be captured. Atipamezole CRI induced a significant (*p* < 0.0001) increase (from 41.8 ± 6.1 bpm to 62.0 ± 20.9 bpm) in heart rate, similar to those reported by IV bolus-administration dogs [15]. However, the heart rate remained below baseline values. It also led to significant (*p* < 0.0001) decreases in systolic, mean, and diastolic blood pressures compared to dexmedetomidine CRI sedative Phases 1 and 2. However, these pressures remained above the hypotensive state (MABP < 60 mmHg) and differed from those reported in cats with transient hypotension [33]. 

When comparing with bolus atipamezole reversal in the medetomidine-sedated dogs, several points can be made concerning the use of CRI atipamezole administration and the current outcome. Firstly, the gradual antagonism of dexmedetomidine may occur with a slow atipamezole administration method (i.e., CRI). Secondly, a smaller dose of atipamezole was used in the current study (50 µg/kg vs. 200 µg/kg) compared to a rapid IV bolus administration of atipamezole in reversing the medetomidine-sedated dogs, which may result in a dose below the optimal level. Thirdly, lingering dexmedetomidine effects might persist, sustaining vasoconstriction and partially explaining the lower-than-baseline heart rate due to residual peripheral vasoconstriction. Additionally, the dexmedetomidine-induced parasympathetic tone could also play a role in contributing to the reduction in heart rate [1,14]. 

The study design employed a decremental CRI dose immediately following an initial high dose of dexmedetomidine, enabling us to capture sufficient sedation and assess behavioral, cardiorespiratory, and EEG characteristics relevant to potential clinical use in dogs. Dexmedetomidine, unlike other general anesthetic drugs, is primarily a sedative, and insufficient sedation can lead to substantial muscle activity artifacts, hindering the study’s main purpose of examining EEG and its clinical utility [22].

In the current study, the sedation achieved was deemed adequate for potential clinical use in sedating dogs for non-invasive procedures. The dogs remained well-immobilized during the CRI phases, as evidenced by minimal EMG and artifact activities. Although each infusion phase lasted only 10 min (Phase 1) to 15 min (Phases 2–4), our clinical experience suggests that dogs without atipamezole treatment may take a while to wake up from the CRI doses used in this study. This prolonged recovery period could result in dogs experiencing hypothermia and a bradycardic state [1,14]. Therefore, we chose the decremental CRI method with atipamezole CRI to study all these dogs. Additionally, the study revealed that the antagonism of dexmedetomidine with atipamezole did not result in rough or delirious recovery in these dogs.

EEG monitoring emerges as highly promising for clinical use in sedation settings, supported by factors such as reduced muscle activity, minimal artifacts, a clear EEG signal during dexmedetomidine sedation, and the ease of electrode application [22]. This promising application aligns with the potential of the CRI regimen, showing promise in clinical situations requiring sedation/immobilization [22,26].

This study has limitations, such as a small sample size and the inclusion of only one breed, specifically male dogs. Basic cardiorespiratory monitoring was used, and the study concentrated on the loading dose for CRI of dexmedetomidine, rather than comparing it to a single loading dose. Despite these limitations, the study successfully demonstrated the EEG patterns of dogs under dexmedetomidine CRI and the antagonism of dexmedetomidine effects with atipamezole CRI in dogs.

## 5. Conclusions

In summary, this study demonstrates that dexmedetomidine CRI induces sedation in dogs, and EEG proves to be a valuable tool for monitoring associated brain-state changes, evident through PSI, DSA, and distinct EEG patterns. The limited analgesic effects associated with dexmedetomidine CRI underscore its primary role as a sedative. Atipamezole is administered to reverse the sedative effects, as indicated by the normalization of PSI, EMG activities, EEG patterns, cardiorespiratory parameters, and awake behavior. Further research is essential to refine and optimize the EEG-guided dexmedetomidine CRI regimen’s application across diverse clinical scenarios, especially when combined with an analgesic drug.

## Figures and Tables

**Table 2:** This table shows the mean (± SD) processed EEG index values in six dogs during different phases of dexmedetomidine CRI. The phases include Phase 0 (awake baseline), Phase 1 (initial dose CRI), Phases 2–4 (maintenance), Phase 5 (atipamezole), and Phase 6 (recovery). Please check the text for more information about experimental phases and drug dosages. In the PSI and SR columns, values with differentletters indicate a statistically significant difference (*p* = 0.0001) between the values.

Phase	PSI	SR (%)	EMG (%)	ARTF (%)	SEF_95_R (Hz)	SEF_95_L (Hz)
0	90.4 ± 4.3 a	0.0 ± 0.0 a	50.6 ± 39.3	21.0 ± 32.8	12.1 ± 7.5	17.2 ± 8.0
1	50.9 ± 30.7 b	8.5 ± 15.5 b	38.2 ± 34.7	18.7 ± 30.1	5.8 ± 3.7	7.5 ± 4.4
2	29.3 ± 18.1 b	7.9 ± 15.2 b	8.7 ± 6.9	14.9 ± 27.1	5.1 ± 2.7	6.1 ± 3.2
3	33.1 ± 19.1 b	9.1 ± 18.4 b	9.5 ± 6.6	7.6 ± 18.4	5.3 ± 3.2	6.1 ± 4.3
4	29.0 ± 18.1 b	19.5 ± 25.6 b	13.8 ± 13.9	4.6 ± 9.5	6.6 ± 4.2	7.2 ± 4.2
5	37.4 ± 24.3 b	14.5 ± 17.7 b	31.7 ± 26	19.3 ± 22.8	6.4 ± 5.3	7.9 ± 5.3
6	42.3 ± 25.8 b	7.8 ± 6.8 b	60.4 ± 17.9	23.3 ± 3.5	4.6 ± 3.1	5.2 ± 3.5

**Table 3:** The mean (± SD) heart rate (HR), systolic blood pressure (SBP), mean blood pressure (MBP), diastolic blood pressure (DBP), respiratory rate (RR), and tolerance to electric stimulation for six dogs during seven phases of dexmedetomidine CRI, followed by atipamezole reversal. Within a column, parameter values with different letters are significantly different (*p* = 0.0001). NA represents-painful stimuli were not administered due to the absence of an analgesic state.

Phase	HR (bpm)	SBP (mmHg)	MBP (mmHg)	DBP (mmHg)	RR (bpm)	Electrical Stimulation (Hz)
0	115.3 ± 36.2 a	160.7 ± 30.4	116 ± 12.7	101.6 ± 1.9	25.0 ± 10.0	NA
1	57.9 ± 33.9 b	182.9 ± 27.3 a	133.7 ± 17.0 a	121.1 ± 18.1 a	17.8 ± 4.9	100.0 ± 0.0
2	39.5 ± 10.9 b	173.8 ± 28.9	131.2 ± 20.4 a	120.3 ± 20.9 a	13.6 ± 3.7	141.7 ± 52.5
3	37.8 ± 3.5 c	158.8 ± 14.8	117.3 ± 11.2	107.3 ± 12.1	12.5 ± 4.2	115.4 ± 30.9
4	41.8 ± 6.1 d	150.7 ± 8.9	111.7 ± 13.9	102.7 ± 13.8	15.1 ± 5.4	100.0 ± 0.0
5	62.0 ± 20.9 e	139.1 ± 22.1 b	100.5 ± 15.8 b	91.3 ± 15.7 b	17.4 ± 5.1	NA
6	76.0 ± 2.8	130.5 ± 6.4	92.0 ± 2.8 b	78.0 ± 2.8 c	22.0 ± 2.8	NA

## Data Availability

Data are contained within the article.
